# Hypothermic cardiac arrest patients admitted to hospital who were not rewarmed with extracorporeal life support: A retrospective study

**DOI:** 10.1016/j.resplu.2023.100443

**Published:** 2023-08-10

**Authors:** Nicolas Hall, Jessika Métrailler-Mermoud, Evelien Cools, Christophe Fehlmann, Pierre-Nicolas Carron, Valentin Rousson, Silke Grabherr, Bettina Schrag, Matthias Kirsch, Vincent Frochaux, Mathieu Pasquier

**Affiliations:** a Department of Emergency Medicine, Lausanne University Hospital and University of Lausanne, Lausanne, Switzerland; b Emergency Service, Hôpital du Valais, Sion, Switzerland; c Acute Medicine Department, Anesthesiology Service, Geneva, Switzerland; d Emergency Department, Geneva, Switzerland; e Center for Primary Care and Public Health (Unisanté), Lausanne, Switzerland; f University Center of Legal Medicine, Lausanne – Geneva, Switzerland; g Lausanne University Hospital and University of Lausanne, Geneva University Hospital and University of Geneva, Switzerland; h Legal Medicine Service, Hospitals Central Institute (ICH), Sion, Switzerland; i Department of Cardiac Surgery, Lausanne University Hospital, Lausanne, Switzerland

**Keywords:** Accidental hypothermia, Cardiac arrest, ECLS, Potassium, HOPE score, Resuscitation

## Abstract

**Aims:**

Our goal was to study hypothermic cardiac arrest (CA) patients who were not rewarmed by Extracorporeal Life Support (ECLS) but were admitted to a hospital equipped for it. The focus was on whether the decisions of non-rewarming, meaning termination of resuscitation, were compliant with international guidelines based on serum potassium at hospital admission.

**Methods:**

We retrospectively included all hypothermic CA who were not rewarmed, from three Swiss centers between 1st January 2000 and 2nd May 2021. Data were extracted from medical charts and assembled into two groups for analysis according to serum potassium. We identified the criteria used to terminate resuscitation. We also retrospectively calculated the HOPE score, a multivariable tool predicting the survival probability in hypothermic CA undergoing ECLS rewarming.

**Results:**

Thirty-eight victims were included in the study. The decision of non-rewarming was compliant with international guidelines for 12 (33%) patients. Among the 36 patients for whom the serum potassium was measured at hospital admission, 24 (67%) had a value that – alone – would have indicated ECLS. For 13 of these 24 (54%) patients, the HOPE score was <10%, meaning that ECLS was not indicated. The HOPE estimation of the survival probabilities, when used with a 10% threshold, supported 23 (68%) of the non-rewarming decisions made by the clinicians.

**Conclusions:**

This study showed a low adherence to international guidelines for hypothermic CA patients. In contrast, most of these non-rewarming decisions made by clinicians would have been compliant with current guidelines based on the HOPE score.

## Introduction

Accidental hypothermia is defined as unintentional reduction of the core temperature below 35 °C.^1^ Vulnerable people usually experience it under circumstances such as cold exposure, substance abuse or trauma.^2^, ^3^, ^4^ In the most severe cases, hypothermia induces impairment of vital functions leading to cardiac arrest (CA).^5^ Hypothermic CA usually appears below 30 °C for healthy adults and below 32 °C for older and multimorbid patients. Extracorporeal life support (ECLS) rewarming, ideally using extracorporeal membrane oxygenation is the method of choice for treating CA secondary to hypothermia.^6^, ^7^ It provides excellent chances of survival with potentially good neurological outcomes.^8^ Historically, the indication to perform ECLS rewarming was based solely on the serum potassium value measured at hospital admission. Recently, the HOPE (hypothermia outcome prediction after ECLS) score has been proposed to guide the decision to use ECLS in hypothermic CA.^7^, ^9^ The HOPE score is based on the following six variables available at hospital admission: age, sex, mechanism of hypothermia, cardiopulmonary resuscitation (CPR) duration, serum potassium, core temperature. It estimates a probability of survival until hospital discharge after ECLS rewarming for hypothermic CA patients. As the HOPE derivation and validation studies were based on retrospective data from consecutive patients rewarmed by ECLS, the group of patients admitted to a hospital who did not receive ECLS rewarming has been little investigated.^10^, ^11^ Our goal was to study a population of hypothermic CA patients who did not receive ECLS rewarming, despite being admitted to a hospital equipped for it. We focused on whether the decision-making processes were compliant with international guidelines prevailing at the time and analysed the decision criteria leading to non-rewarming decisions. We also analysed the post-mortem observations, when available, and retrospectively calculated the estimated survival probabilities of the patients by using the HOPE score.

## Methods

### Study population

We conducted a retrospective observational study from data collected from three participating Swiss centres: University Hospital of Lausanne, Geneva University Hospitals and Hôpital du Valais in Sion. We included all patients older than 18 years with hypothermic CA at hospital admission who did not receive ECLS rewarming, indicating termination of resuscitation. The inclusion period was January 1, 2000, to May 2, 2021. To exclude any primary cause of CA other than hypothermia, we defined 32 °C as the upper threshold of core temperature below which CA could potentially be attributed to hypothermia.^2^, ^12^, ^13^, ^14^ We excluded patients who were not in CA at hospital admission, those who received ECLS rewarming and all patients under the age of 18 years.

### Data collection

The following data were extracted by a single reviewer from the included patients’ prehospital and hospital charts: age, sex, mechanism of hypothermia, presence of noticeable signs of trauma, whether the CA was witnessed or not, CPR duration until hospital admission, CA rhythm and core temperature measured at hospital admission. The mechanism of hypothermia was classified into two distinct categories: asphyxia-related (avalanche victims with head fully covered by water or snow and in CA at extrication or submersion in water) and non-asphyxia-related (cold exposure or immersion).^15^ The CPR duration used for the HOPE score was calculated from the initiation of CPR until the time of hospital admission plus 20 min, a delay that we considered to be equivalent to the assumed time needed to start ECLS.^7^, ^10^ We extracted the following biological parameters: serum potassium, lactate concentration, pH, PaCO_2_ and end-tidal CO2. In emergency department reports, we specifically collected the decision criteria given by physicians leading to the decision not to use ECLS techniques, and therefore stop the resuscitation. The University Center of Legal Medicine of Lausanne – Geneva and the Forensic Medicine Institute of Sion provided us with forensic autopsy reports, as well as anatomic, pathological and radiological diagnoses of the included patients.

The study and data collection were approved on September 9, 2021, by the local ethics committee “Commission cantonale d’éthique de la recherche sur l’être humain – Vaud (CER-VD)”, Switzerland (N° 2021-01637).

### Primary outcome

Our primary study outcome was to assess the compliance of the decision not to rewarm patients, based on the potassium level, as mentioned by the international recommendations prevailing at the time of hospital admission. According to the guidelines available from 2001 to 2021, a potassium level higher than 12 mmol/L contraindicated ECLS rewarming.^2^, ^13^, ^16^, ^17^ Regarding avalanche victims, guidelines have evolved with a maximum threshold for rewarming set at 12 mmol/L from 2001 to 2012^18^ and 8 mmol/L from 2013 to 2021.^17^ As incorporated in the 2021 recommendations, the HOPE score was not used in the decision-making process regarding ECLS for the included patients.

### Secondary outcomes

Our secondary outcomes were the analysis of the criteria leading to decisions not to qualify patients for ECLS rewarming for victims whose potassium value did not contraindicate the use of ECLS, and the retrospective calculation of the HOPE survival probabilities by using the dedicated website www.hypothermiascore.org*.* We also retrospectively assessed the influence that would have had the HOPE score by comparing the indication for ECLS rewarming with either the potassium criterion or the estimation of the survival probabilities provided by HOPE. We considered a HOPE-estimated survival probability of <0.1 as a criterion that would contraindicate ECLS rewarming. The 0.1 threshold was chosen because it corresponds to that used in the external validation study of the HOPE score.^9^, ^11^ Finally, we analysed the post-mortem forensic and autopsy reports for cause of death.

### Statistical analysis

The data extracted were stored on an Excel spreadsheet (Microsoft, Redmond, WA, USA) and exported to Stata version 20 (Stata Corporation, College Station, TX, USA) for statistical analysis. We presented normally distributed continuous variables as mean ± standard deviation and compared them by using Student’s t-tests. Non-normally continuous variables were presented as medians and interquartile ranges and compared with Mann-Whitney’s U tests. We expressed categorical data as numbers and percentages and compared them with Pearson’s chi-squared test or Fisher’s exact test as appropriate. Statistical significance was considered for a two-tailed p-value of <0.05.

## Results

**Fig. 1:**
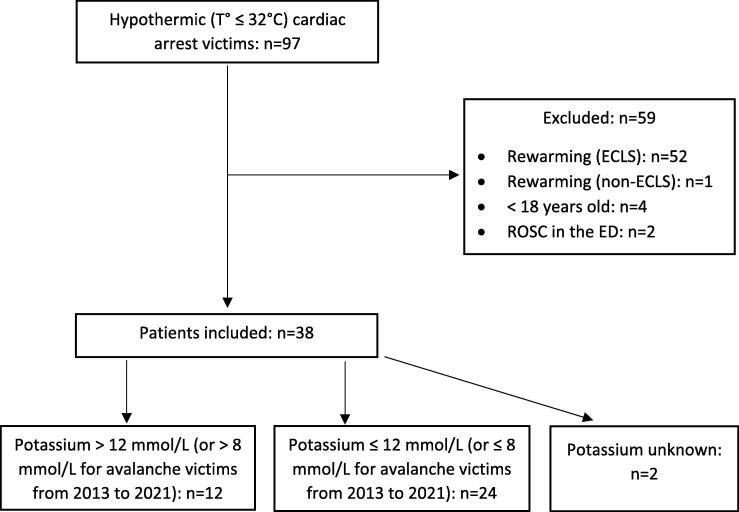
Flowchart of patient inclusion. Patients with hypothermic cardiac arrests at hospital admission (January 1, 2000, to May 2, 2021). Abbreviations: ECLS, extracorporeal life support; ED, emergency department; ROSC, return of spontaneous circulation.

Among the 97 hypothermic CA patients identified, 38 fulfilled the inclusion criteria after selection according to the exclusion factors described earlier. Three patients were excluded because of the return of spontaneous circulation in the emergency department (*n* = 2) or death following rewarming attempts with the use of a non-ECLS rewarming technique (*n* = 1) (Fig. 1). Twenty-eight (74%) victims were male. The median age was 55 years (range 18–94, interquartile range 40–68). Fourteen patients were avalanche victims. The characteristics of the study population are presented in Table 1, according to the potassium triage criterion. Serum potassium levels were unavailable at hospital admission for two patients.

**Table 1:** Characteristics of the study population at hospital admission according to the serum potassium triage criterion. Abbreviations: CA, cardiac arrest; CPR, cardiopulmonary resuscitation; ETCO_2_, end-tidal carbon dioxide; HOPE, (hypothermia outcome prediction after extracorporeal life support); IQR, interquartile range; PaCO_2_, partial pressure of carbon dioxide; PEA, pulseless electrical activity; VF, ventricular fibrillation.

	Total (*n* = 38)^a^	K^+^ > 12 mmol/L (*n* = 12)	K^+^ ≤ 12 mmol/L (*n* = 24)	*P*
Males, n (%)	28 (74)	11 (92)	16 (67)	0.22
Median age, years (IQR)	55 (40–68)	46 ± 14	55 ± 21	0.19
Mechanism, n (%)				
Asphyxia	22 (58)	7 (58)	15 (63)	0.81
No asphyxia	16 (42)	5 (42)	9 (38)	
Median CPR duration, min (IQR)	104 (75–125)	117 (85–164)	92 (74–123)	0.28
Not documented, n (%)	3 (8)			
Core temperature, °C (mean ± SD)	25 ± 6.2	23 ± 9.5	26 ± 4.5	0.27
Not documented, n (%)	2 (5)			
CA rhythm, n (%)				
Asystole	29 (76)	11 (100)	17 (71)	0.045
VF, PEA	7 (18)	0 (0)	7 (29)	
Not documented	2 (5)			
Trauma, n (%)				
Absent	29 (76)	12 (100)	16 (67)	0.03
Serum potassium, mmol/L (mean ± SD)	9.8 ± 5.3	16 ± 2.9	6.7 ± 3.0	-
Not documented, n (%)	2 (5)			
HOPE estimated survival probabilities, n (%)				
HOPE <10%	23 (60)	10 (100)	13 (54)	0.009
HOPE ≥10%	11 (29)	0 (0)	11 (46)	
Not documented	4 (11)	2	0	
pH, (mean ± SD)	6.71 ± 0.28	6.60 ± 0.40	6.75 ± 0.24	0.13
Not documented, n (%)	14 (37)			
Lactate concentration, mmol/L, (mean ± SD)	19.7 ± 8.0	25.3 ± 7.8	17.9 ± 7.3	0.02
Not documented, n (%)	10 (26)			
PaCO_2_, mmHg, median (IQR)	80 (59–127)	111 (67–168)	77 (56–107)	0.28
Not documented, n (%)	10 (26)			
ETCO_2_, mmHg, median (IQR)	7 (2–27)	27 (7–30)	3.5 (2–5)	0.38
Not documented, n (%)	30 (79)			
Witnessed CA, n (%)	4 (10)	0 (0)	2 (8)	0.54

a The serum potassium value was unavailable at hospital admission for two patients.

The decision to not rewarm patients was compliant with the international guidelines for 12 (33%) patients, meaning that their potassium value was above the maximum threshold that would indicate ECLS rewarming. Among the 36 patients for whom the serum potassium was measured at hospital admission, 24 (67%) had a serum potassium value that, alone, would not have contraindicated ECLS. The criteria leading to the decision of non-rewarming and resuscitation interruption for these 24 patients are presented in Table 2. A combination of several criteria resulted in a non-rewarming approach for 14 patients. The decision was documented in the medical chart as a multidisciplinary medical agreement in 13 of 24 cases.

**Table 2:** Criteria mentioned in the medical chart and leading to decisions of non-rewarming for the 24 victims whose potassium value at hospital admission did not contraindicate rewarming according to guidelines. Abbreviations: CA, cardiac arrest; CPR, cardiopulmonary resuscitation; HOPE, hypothermia outcome prediction after extracorporeal life support.

Criteria leading to the decision of non-rewarming^a^	*n*
Variables included in the HOPE estimation of survival probabilities	
Core temperature insufficiently low to cause CA by pure hypothermia (temperature values in °C: 29.6, 30.1, 31, 31.4)	4
Potassium measurement at hospital admission (measurements in mmol/L: 6.8, 10.1, 10.2)	3
Long duration of CPR (in minutes: 55, 68, 158)	3
Old age (83)	1
Drowning assumed to be a more likely cause of CA than hypothermia based on circumstances	1
Variables not included in the HOPE estimation of survival probabilities	
Overall biological parameters of arterial blood-gas measurements (lactate concentration, pH, potassium)	7
Duration of no-flow^b^ (in minutes: 15, 20, 30, unknown *n* = 2)	5
Asystole as CA rhythm	4
Overall prognosis and comorbidities	3
Do not resuscitate order or presumed wishes stated by relatives	3
Other cause of CA more likely than hypothermia based on circumstances (heart disease, head trauma)	3
Duration of burial for avalanche victims (short burial, i.e. <35 min, durations in minutes unknown)	2
Undetermined^c^	4

a Multiple decisional criteria described in the table may apply to the same patient.

b No-flow is the time during which cardiac output is absent, before any CPR is performed

c No criteria for non-rewarming were identified in the patient’s medical records for these cases.

**Fig. 2:**
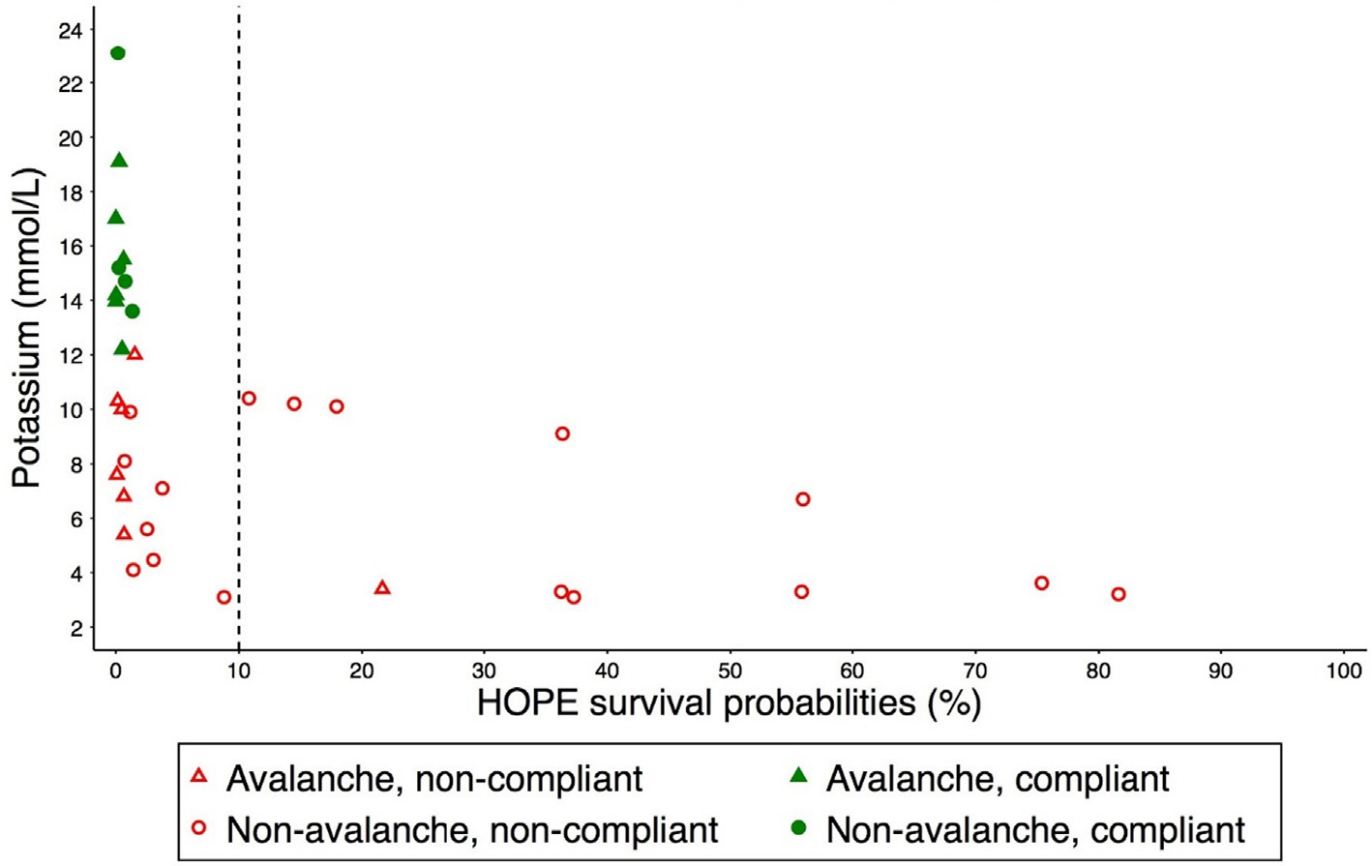
Probabilities of survival according to the HOPE score and potassium values at hospital admission of 34 patients with hypothermic cardiac arrest who were not rewarmed with ECLS. Compliance with guidelines refers to non-rewarming of patients with a potassium value >12 mmol/L for non-avalanche victims and >12 mmol/L (2001–2012) or >8 mmol/L (2013–2021) for avalanche victims. No included avalanche patient from 2013 to 2021 had a potassium value between 8 and 12 mmol/L. The vertical dashed line corresponds to the 10% threshold value of HOPE, a value <10% suggesting that ECLS rewarming would not be indicated. Abbreviations: ECLS, extracorporeal life support; HOPE (hypothermia outcome prediction after ECLS).

**Fig. 3:**
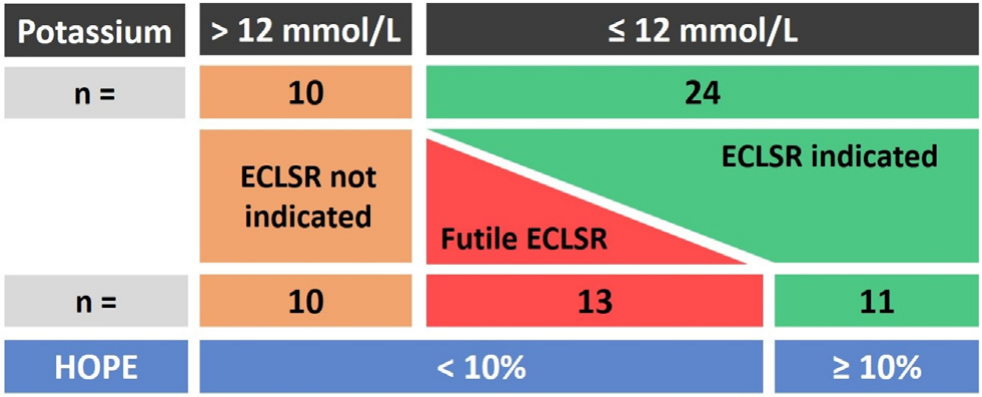
Distribution of the study population regarding eligibility for ECLS rewarming and comparison between potassium value alone (cut-off at 12 mmol/L) and HOPE (cut-off at 10%) as triage tools. Abbreviations: ECLSR, extracorporeal life support rewarming; HOPE, hypothermia outcome prediction after ECLS.

The HOPE score was calculated for 34 (89%) of the 38 included patients. The remaining four patients had at least one parameter missing in their medical chart, which made the HOPE calculation not feasible. The HOPE estimation of the survival probabilities was <0.1 for all 10 patients with a potassium value higher than 12 mmol/L. The HOPE score was <0.1 in 13 of the 24 (54%) who had a serum potassium value that, alone, would not have contraindicated ECLS (Fig. 2). In total, the HOPE estimation of the survival probabilities, when used with a 0.10 threshold, supported 23 (68%) of the non-rewarming decisions made for the included patients, and does not support the non-rewarming decisions for the remaining 11 patients (32%) (Fig. 3).

Post-mortem external examinations and autopsies were respectively performed for 24 and 13 victims. A summary of the findings is presented in Table 3**.** Among the five patients who presented one typical hypothermia-related finding such as Wischnewski spots, frost erythema, frostbite, bright red lividity or bloody discoloration of synovial fluid,^19^ two had a definite diagnosis of death by accidental hypothermia. Three other patients had circumstantial evidence of cold exposure without any other identified cause of death. Six of these combined eight patients (75%) had a HOPE score of ≥0.1 and none had evidence of asphyxia. The autopsies revealed a potential death by accidental hypothermia for only one victim among those with a HOPE score of <0.1. All nine victims with a presumed death by drowning according to circumstances had a potassium value lower than 12 mmol/L. Three (33%) of them had confirmation of drowning as the cause of death at the autopsy.

**Table 3.** Results and conclusions of the forensic post-mortem investigations which were available for 24 (63%) of the included patients. Abbreviations: ECLS, extracorporeal life support; HOPE, hypothermia outcome prediction after ECLS.

**Detailed findings from post-mortem investigations including external examination and autopsy**	**n**
**Type of forensic investigations**	**24**
External examination	24
Autopsy	13
Additional forensic imaging^a^	9
**Confirmed causes of death**	**24**
Drowning	3
Mechanical asphyxia (chest compression impairing ventilation, snow burial in avalanches)	3
Accidental hypothermia	2
Asphyxia by non-viable air composition	2
Trauma (severe craniocerebral injury)	1
Undetermined	13
**Hypothermia-related findings**^b^	**10**
Wischnewski spots^c^	3
Reddish-purplish ecchymotic discoloration of the knee^d^	3
Pink-red lividities	2
Cherry red liquid blood	1
Frostbite lesions	1
**No hypothermia-related signs**	**17**
**Major findings at autopsies and external examination**	
Acute pulmonary oedema	9
Visceral blood stasis	7
Pulmonary emphysema	6
Cerebral oedema	3
Fractures (femur, cranial, costal, dental)	3
Petechiae (conjunctival, interventricular septum, bladder, palpebral, oral mucosal)	3
**Findings for victims with potassium values and HOPE scores in favour of ECLS rewarming (< 12 mmol/L and HOPE > 10%)**	**10**
Presence of trauma (fracture, subdural haematoma)	3
Asphyxia-related death (snow-burial avalanche, mechanical asphyxia)	2
Presence of hypothermia-related signs (Wischnewski spots a, red lividities, ecchymotic areas on knees b)	4
Undetermined causes of death	6

^a^ We consider radiological imaging as an integral part of an autopsy.

^b^ Multiple hypothermia-related signs may apply to the same patient.

^c^ Wischnewski spots: foci of petechial haemorrhages of the gastric and duodenal mucosa with ulcerations containing thrombotic haemorrhagic material.

^d^ This description could possibly refer to “frost erythema”.

## Discussion

To the best of our knowledge, this study is the first to specifically address hypothermic CA patients admitted to a hospital equipped for ECLS rewarming, but who did not receive it. The adherence to guidelines regarding resuscitation was low. Only 33% of non-rewarming decisions were compliant with guidelines that use potassium as the triage criterion.^12^, ^13^ Twenty-four patients (67%) had a potassium value that –alone –would have not contraindicated ECLS rewarming. Various alternative clinical, biological, prognostic or resuscitation-related criteria were mentioned in the medical charts to support the decision to terminate resuscitation.

The 33% rate of adherence to guidelines should be interpreted with caution. The level of evidence underlying the use of the potassium level alone to decide whether to rewarm a hypothermic CA patient or not is low.^6^ Evidence is based mainly on observational studies, case reports, and retrospective case series with small study populations.^2^, ^6^, ^20^, ^21^ The present study highlights the integration of the whole clinical context into the decision-making process led by clinicians. The analysis of the arguments used to support the decision not to reanimate a patient is therefore interesting, especially for patients with a potassium value of ≤12 mmol/L.

Unfavourable arterial **blood gas values** (high lactate concentration and low pH) were mentioned as information that led to resuscitation termination decisions in 7 (29%) of the 24 victims whose potassium level would have indicated ECLS rewarming. Low initial lactate concentration and high pH are associated with survival and good neurological outcome after ECLS rewarming for hypothermic CA.^22^, ^23^ A positive correlation between lactate and potassium concentrations has also been reported.^23^, ^24^ In the present study, the lactate concentration was also significantly lower (*p* = 0.02) in patients with a potassium of ≤12 mmol/L. In a multivariable analysis, pH and lactate concentration were not found to be independently associated with survival and the authors warned about the inappropriate use of biological parameters for ECLS rewarming decisions.^8^ The HOPE score derivation study did not include lactate concentration as a potential predictor, as information about lactate concentration was unfrequently available.^10^ The additional value of lactate concentrations to the already used predictive variables included in the HOPE score may be studied in the future.

Four victims did not receive ECLS rewarming because of their long or unknown **no-flow** duration. No-flow duration is integrated into the decision process to terminate normothermic CA resuscitation.^25^, ^26^ However, several studies have shown hypothermic CA survival with good neurological outcome despite no-flow durations exceeding 1 h.^27^, ^28^ The majority of hypothermic CAs are unwitnessed, meaning that the duration of no-flow is unknown, as was observed in the present study. ECLS rewarming should be considered even for long or unknown no-flow durations.^29^

**Asystole** was mentioned as a criterion leading to the decision of non-rewarming in four patients. Twenty-nine victims (76%) had asystole as the first cardiac rhythm measured at hospital admission, including all victims with a potassium value >12 mmol/L. This is approximately twice as high as reported in other studies of hypothermic CA.^1^, ^30^ However, none of the patients included in our study were rewarmed or declared eligible for ECLS rewarming at the time, presumably indicating more critical cases. We focused on the first rhythm measured at the hospital in order to estimate the probability of in-hospital survival with HOPE. It does not reflect the initial CA rhythm, as only two patients included had a witnessed CA. Asystole is associated with poorer survival and poorer neurological outcome for a hypothermic CA but interestingly not independently. It is not systematically a progression of an initial shockable rhythm in this context.^31^ Asystole is therefore no longer considered an exclusionary factor for ECLS rewarming.^9^

**Trauma** was the reason to stop resuscitation for two victims with a potassium of ≤12 mmol/L. Trauma might be challenging to detect in the case of a hypothermic CA, in particular for avalanche victims.^32^ It should be considered as a reversible cause of CA until proven otherwise.^33^ In clinical practice, the performance of an eFAST (extended focused assessment with sonography in trauma) ultrasound examination during CPR could help orient the diagnosis. Concomitant trauma such as severe brain injury is no longer an absolute contraindication to ECLS rewarming,^9^ as survival from polytrauma and hypothermic CA has been reported.^34^

Although 58% of the included victims experienced **asphyxia** (through snow burial in avalanches or drowning), it was the reason reported for discontinuing resuscitation and ineligibility to receive ECLS rewarming for only one patient. Regardless of the mechanism of occurrence, asphyxia leads to hypoxic ischaemic brain injuries and CA mostly through hypoxemia and hypercapnia.^7^, ^35^ Avalanche victims predominantly die of asphyxia and trauma.^33^, ^36^, ^37^, ^38^ In this context, guidelines for ECLS rewarming of avalanche victims with CA have been adapted historically through the successive lowering of the upper threshold of measured potassium levels, reflecting their poorer outcomes (most suffering from asphyxia-induced CA) compared with patients with pure hypothermia.^12^, ^13^, ^17^, ^18^, ^20^, ^39^, ^40^ Interestingly, potassium thresholds were not adjusted in the context of accidental hypothermia and drowning, despite similar mechanisms. Asphyxia may not be the cause of CA in avalanche or drowning victims, notably if breathing under the snow was enabled by the presence of an air pocket,^33^ or if immersion occurred before submersion in drowning.^41^ In the case of unclear circumstances, a non-asphyxia mechanism should be preferred to calculate the HOPE score in order to minimise the risk of underestimating the probability of survival after ECLS rewarming.

For eight (33%) victims whose potassium value was in favour of ECLS rewarming, several **variables used to calculate the HOPE score** were mentioned as criteria leading to non-rewarming, namely a core temperature insufficiently low to induce hypothermic CA, a long CPR duration, advanced age and a mechanism of asphyxia in the case of drowning. The assessment and discrimination of patients eligible for ECLS was improved by the integration of these five predictors, in addition to the potassium level to constitute the HOPE score, compared with the use of potassium level alone. The areas under the curve in the derivation and validation studies of HOPE are 0.866 and 0.825, respectively, which are indeed both superior to 0.774 for potassium alone, which remains below the threshold of 0.8, implying excellent discrimination.^10^, ^11^ The HOPE score would have supported the clinicians’ decision in most cases, as 68% of the victims in our study would have not been rewarmed, if a HOPE-estimated survival probability of <0.1 was used as a criterion not to rewarm the patient.

However, the HOPE score would have contraindicated ECLS for 13 (54%) of the 24 victims whose potassium value at hospital admission suggested ECLS rewarming. Our results confirm that the use of HOPE improves triage by limiting the number of patients with futile rewarming compared with the use of potassium levels alone. Recall also that the HOPE score has good calibration and excellent discrimination, meaning that its use could be reproducible in a similar case-mix of patients. The positive predictive value (PPV) is the proportion of patients who survived after ECLS rewarming among those with a HOPE score of ≥0.1. The PPVs were 55% and 57%, respectively, in the derivation and validation studies of HOPE. Given the similar scattered distribution of patients, we extrapolated the PPV to our population and concluded that 6 of the 11 patients with a HOPE score of ≥0.1 could have benefited from ECLS rewarming with potential survival. Post-mortem external examinations and autopsies were performed on 10 and 6 of these victims respectively. They revealed one death by accidental hypothermia, one by head trauma and the remaining 8 by undetermined cause, i.e. hypothermia not excluded.

Of all patients who had post-mortem investigations, two had accidental hypothermia as the cause of death. One of them had a HOPE score of <0.1, meaning that according to current guidelines, ECLS rewarming would not have been indicated either. At some point, hypothermia is overcome and becomes irreversible in the case of pure hypothermia-induced CA, making the chances of survival after ECLS rewarming negligible. The use of the HOPE score is relevant in this specific context, as it discriminates patients who have crossed this threshold and are no longer eligible for ECLS rewarming.

## Limitations

An important limitation is the retrospective design of the study. All data were extracted by a single reviewer from medical charts, meaning that the results relied on the quality of the documentation. Another limitation is the sample size of our study due to the paucity of eligible patients over the last 20 years and that some archived patient records were no longer available.

## Conclusions

The adherence to guidelines regarding resuscitation of hypothermic CA patients admitted to a hospital equipped for ECLS rewarming but who did not receive it was poor, as 67% of them had a potassium value that - alone - would not have contraindicated ECLS rewarming. Multivariable tools have recently been recommended to replace the use of potassium levels alone to guide decisions of ECLS rewarming for hypothermic CA patients. The HOPE estimation of the survival probabilities, when used with a 0.1 threshold, would have supported 68% of the non-rewarming decisions made by the clinicians.

## Funding source

This research received no external funding. The article processing charges were funded by the Lausanne University Open Access program.

## Declaration of Competing Interest

The authors declare that they have no known competing financial interests or personal relationships that could have appeared to influence the work reported in this paper.
